# 51. A case of anti-synthetase syndrome with subsequent CMV infection

**DOI:** 10.1093/rap/rky034.014

**Published:** 2018-09-20

**Authors:** Asif Nawaz, Julie Barber, Yamin Rashid

**Affiliations:** 1 Acute Medicine & Rheumatology, Withybush General Hospital, Haverfordwest, UNITED KINGDOM; 2 Rheumatology, Withybush General Hospital Haverfordwest, Haverfordwest, UNITED KINGDOM

**Introduction:** A 43 year old male presented with history of shortness of breath, proximal muscle weakness, and pompholyx. He had a positive ENA, positive Jo-1 antibodies, raised CK. High-resolution computed tomography (HRCT) and VATS biopsy showed nonspecific interstitial fibrosis. These findings along with clinical features and investigations confirmed the diagnosis of antisynthetase syndrome (ASS).

**Case description:** A 43 year old male with no significant past medical history was referred to rheumatology clinic with complaints of muscle weakness, shortness of breath on exertion, muscle aches particularly in his knees and generalised malaise. His exercise tolerance had significantly reduced from a normal active lifestyle to being unable to climb tanks as part of his work as an engineer on an oil refinery. He denied any chemical or asbestos exposure as part of his job. He was an ex-smoker (20 pack year’s history), no alcohol use, no known allergies and did not take any regular medications. Physical examination revealed grade 1 clubbing; examination of the chest was unremarkable except the localised area of bronchial breathing in right base. Cardiovascular and abdominal examinations were unremarkable. On neurological examination, the only positive finding was proximal muscle weakness in the thighs. Routine blood tests including FBC, U&Es, LFT’s were normal. His erythrocyte sedimentation rate was 19 (0-15 mm/hr) and C-reactive protein was normal. Creatine kinase (CK) levels were elevated at 575 (0-20 mg/L). Serum ferritin urinalysis, hepatitis serology, HIV screen were unremarkable. He had a negative rheumatoid factor (<20.0) but positive ENA result with positive anti-Jo-1 antibodies (negative for anti-La, anti-RNP, and anti-Sm antibodies). ANA, ANCA, RNP, and scl-70 were negative. C3, C4 and immunoglobulin level were normal. A high resolution computed tomography (HRCT) scan showed reticular opacities bilaterally in the subpleural areas, at the level of both lower lobes, dorsally and minor similar changes at the level of the right upper lobe, anterolaterally. Discrete patchy ground-glass opacities seen at the dependent areas, in both lung bases. Overall appearances were most in keeping with non-specific interstitial pneumonitis (NSIP). The diagnosis of ASS in this patient was established on the basis of the history of pompholyx in the past (although he had no hyperkeratosis on the hand), Positive Anti Jo-1, NISP, myositis and raised CK. This patient was started initially on prednisolone and methotrexate following which he responded very well and his CK dropped down to normal, two years later he developed changes on HRCT suggestive of methotrexate-induced pneumonitis and methotrexate was withdrawn. He was treated with immunoglobulin infusion and steroid, but lung function tests continued to deteriorate. In view of this, he underwent VATs biopsy which showed NSIP pattern. He had cyclophosphamide in six cycles then MMF (mycophenolate mofetil) as maintenance therapy and advice from a center of excellence. Lung function tests improved significantly. Four years after presentation, he developed life-threatening CMV pneumonitis whilst on maintenance MMF 1g BD (commenced 22 months prior) and prednisolone 10mg once daily and was intubated and ventilated and required valganciclovir. HIV testing and testing for *Pneumocystis carinii* were negative. Subsequent testing showed an inadequate response to pneumonia and tetanus vaccinations and he developed herpetic lesions whilst on MMF 500mg daily and prednisolone 10mg daily and increasing immunosuppression was thought inadvisable. The recent development of EMG pattern consistent with myositis with a rise in total CK to 563 U/L has been treated with dose MMF and immunoglobulin infusion six-weekly. His lung function test in 2012 showed FEV1 3.88(91%), the FVC 4.43 (86%) Kco 85%, and TLco of 66< in 2012 while in December 2014 these figures were FEV1 4.16(99%) FVC 4.89(95%) Kco64%, and TLco of 52%. His last lung function test performed in September 2017 were FEV1 3.64 (89%) FVC 4.4 (88%) Kco 78%. Myositis improved significantly and he now cycles for 50 miles/week regularly.

**Discussion:** ASS is an immune-mediated disorder which is very rare (prevalence of 1.5 per 100.000 populations). Hallmark features consist of the combination of interstitial lung disease (ILD), myopathy, fever, and polyarthralgia. The autoimmune screen includes anti-aminoacyl-transfer RNA synthetase positive more frequently that of anti-Jo-1 antibody which is present in approximately 80% of cases. Other less frequent antibodies in ASS are anti-PL-7 and anti-EJ.ILD is being reported in 80-90% of cases of ASS and lung involvement is thought to be the most important prognostic indicator. NSIP is the most frequently reported histological pattern in ASS and the presence of fibrosis is an adverse predictor for response to immunosuppressive therapies.Myositis is reported in 30-60% of ASS patients and anti-Jo-1 positivity can pre-date clinical myositis. Most treatment regimens for ASS describe a combination of glucocorticosteroids and other immunosuppressants such as cyclophosphamide with pulmonary disease response dictating treatment duration.

**Key Learning Points:** Mycophenolate mofetil is increasingly used and experience in the renal transplant field suggests that although primary CMV infection is not more frequent than in other renal transplant patients not on MMF. The infection is more likely to be symptomatic. Rheumatologists should be alert to this potential diagnosis in patients treated with MMF. In summary, ASS is a rare systemic autoimmune condition characterized by a combination of ILD, myopathy, fevers, and polyarthralgia. HRCT typically shows an NSIP pattern and may help to suggest the diagnosis in a patient presenting with appropriate clinical features. Atypical infection may complicate the disease course when immunosuppressive agents are used.

**Disclosure: A. Nawaz:** None. **J. Barber:** None. **Y. Rashid:** None.

